# Anti-Biofilm Efficacy of Commonly Used Wound Care Products in In Vitro Settings

**DOI:** 10.3390/antibiotics12030536

**Published:** 2023-03-08

**Authors:** Matthew Regulski, Matthew F. Myntti, Garth A. James

**Affiliations:** 1 Wound Care Institute of Ocean County, 54 Bey Lea Road, Toms River, NJ 08753, USA; 2 Next Science^®^ LLC, 10550 Deerwood Park Blvd, Suite 300, Jacksonville, FL 32256, USA; 3 Center for Biofilm Engineering, Montana State University, 366 Barnard Hall, Bozeman, MT 59717, USA

**Keywords:** biofilm, biofilm disruption, wound gel, silver dressings, collagenase, chronic wounds, wound infection, in vitro biofilm models

## Abstract

Considering the prevalence and pathogenicity of biofilms in wounds, this study was designed to evaluate the anti-biofilm capabilities of eight commercially available wound care products using established in vitro assays for biofilms. The products evaluated included dressings with multiple delivery formats for ionic silver including nanocrystalline, gelling fibers, polyurethane (PU) foam, and polymer matrix. Additionally, non-silver-based products including an extracellular polymeric substance (EPS)-dissolving antimicrobial wound gel (BDWG), a collagenase-based debriding ointment and a fish skin-based skin substitute were also evaluated. The products were evaluated on *Staphylococcus aureus* and *Pseudomonas aeruginosa* mixed-species biofilms grown using colony drip flow reactor (CDFR) and standard drip flow reactor (DFR) methodologies. Anti-biofilm efficacy was measured by viable plate counts and confocal scanning laser microscopy (CSLM). Four of the eight wound care products tested were efficacious in inhibiting growth of new biofilm when compared with untreated controls. These four products were further evaluated against mature biofilms. BDWG was the only product that achieved greater than 2-log growth reduction (5.88 and 6.58 for *S. aureus* and *P. aeruginosa*, respectively) of a mature biofilm. Evaluating both biofilm prevention and mature biofilm disruption capacity is important to a comprehensive understanding of the anti-biofilm efficacy of wound care products.

## 1. Introduction

Wound biofilm formation can begin through ubiquitously present, endogenous, or exogenous microbes that attach to the wound surface and proliferate [1]. Under ordinary circumstances, the host immune system can fight bacterial growth from free-floating or planktonic bacteria [1,2,3]. However, in an immunocompromised patient or when bacterial growth is uninhibited, the bacteria multiply and build a complex community protected by a matrix of extracellular polymeric substances (EPS) known as the biofilm matrix [4,5,6]. The presence of biofilm is medically recognized as a leading cause of chronicity, with the Center for Disease Control and Prevention (CDC) estimating that biofilms are responsible for over 65% of all chronic bacterial infections, while the National Institutes of Health (NIH) estimating it at around 80% of microbial infections [7,8].

Polymicrobial biofilms are pervasive in most chronic wounds. Mixed-species bacterial communities encased within the EPS exhibit intrinsic tolerance to antibiotics, antiseptics, and antimicrobials [9,10,11,12]. Biofilms also exhibit varied defense mechanisms against environmental stresses and host immune responses and engender secretion of inflammatory mediators that can impede the natural wound healing cascade while sustaining the biofilm [13,14,15,16,17,18]. Research indicates that the key to treating a biofilm is to break down the protective EPS, a structurally strengthened complex of cross-linked polysaccharide polymers linked with metallic ions and containing microbial and host proteins and nucleic acids [19,20,21,22,23,24,25,26,27,28,29,30,31].

Despite the accepted pathogenicity of biofilms in wounds, there are limited objective studies assessing the efficacy of commonly used wound treatment products on preventing biofilm formation, as well as disrupting mature biofilm. This in vitro study sought to facilitate an understanding of the anti-biofilm efficacy of commonly used wound care products using established assays and techniques for biofilm prevention/inhibition and mature biofilm treatment/disruption [32,33,34,35,36]. Mixed-species biofilms are more common than single-species biofilms in chronic wounds, with *S. aureus* and *P. aeruginosa* being the most common [37,38,39,40]. Mixed species biofilms with these two species were thus utilized in this study to simulate the prevention and treatment of polymicrobial biofilm wound infections. Given that antimicrobial silver-based wound dressing products account for almost two-thirds of the antimicrobial wound dressing market with an estimated compound annual growth rate of 12.2% between 2022 and 2029, several advanced silver wound dressing products were included for evaluation [41,42,43]. Additionally, non-silver wound care products with differing mechanisms of action including an EPS disrupting wound gel with antimicrobial activity, an enzymatic collagenase wound debriding product and a fish skin graft product theoretically lacking antimicrobial and EPS disrupting activity were also tested to minimally address the spectrum of wound care products in use. A total of eight commercially available wound care products were tested. All the products tested are either FDA cleared, or FDA approved for use and do not represent an exhaustive list of commercially available wound care products available in the wound care market.

## 2. Results

### 2.1. Efficacy of Wound Care Products in Preventing the Formation of New Biofilm in a CDFR Determined by Viable Plate Count

The mixed-species biofilm, grown using the CDFR methodology and analyzed by viable plate count methodology indicated that several of the products evaluated were able to impact the formation of biofilm in a statistically significant manner when compared with the untreated control.

**Figure 1:**
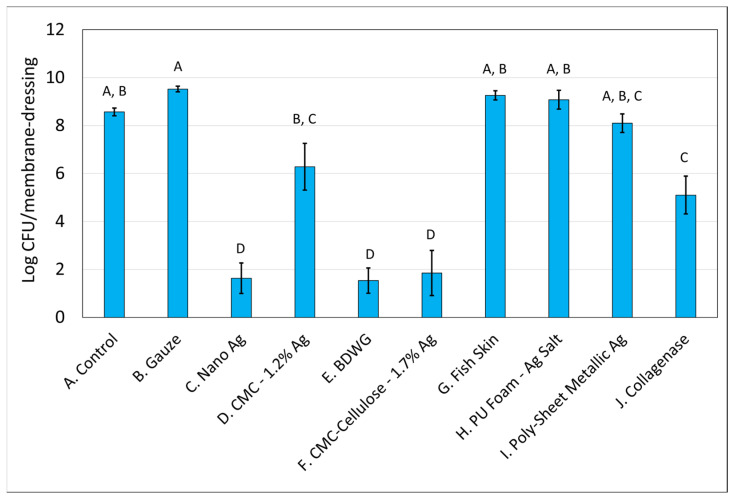
*S. aureus* Log CFU recovered from membranes inoculated with mixed bacterial species and either left untreated (A), treated with saline soaked gauze (B), or treated with wound care products (C through J). If treatments have the same letter listed above the column, they are not statistically different at *p* = 0.05. The error bars depict the standard error of mean.

**Figure 2:**
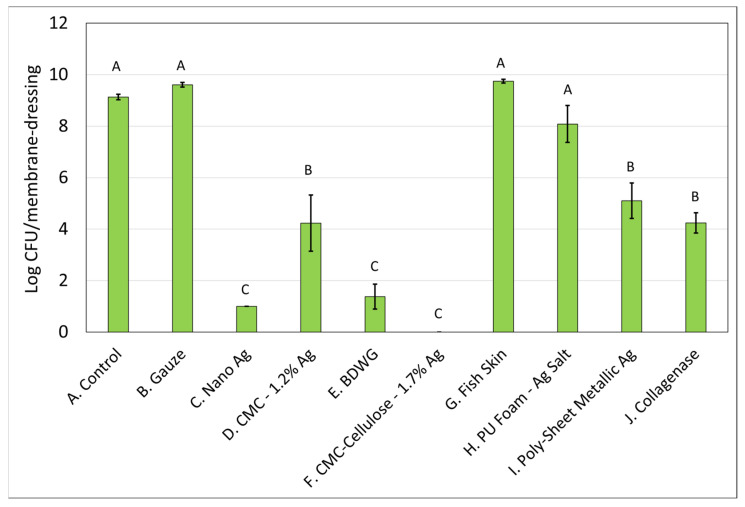
*P. aeruginosa* Log CFU recovered from membranes inoculated with mixed bacterial species and left untreated (A), treated with saline soaked gauze (B) or treated with wound care products (C through J). If treatments have the same letter listed above the column, they are not statistically different at *p* = 0.05. The error bars depict the standard error of mean.

Figure 1 and Figure 2 demonstrate the recovered colony forming units (CFU) for *S. aureus* and *P. aeruginosa*, respectively, from membranes either left untreated (control), treated with saline soaked gauze, or treated with wound care products and treatment efficacy determined by viable plate counts. A Tukey’s *t*-test of the treatments determined that Nano Ag, BDWG, CMC-Cellulose-1.7% Ag, and Collagenase were able to impede new biofilm growth of both bacterial species in a statistically significant manner when compared with the untreated control. CMC-1.2% Ag and Poly-Sheet Metallic Ag were only able to inhibit *P. aeruginosa* biofilm growth in a statistically significant manner when compared with untreated controls in the mixed-species biofilm prevention assay (*p* < 0.05). Saline-soaked gauze, Fish Skin and PU Foam-Ag Salt were comparable to the untreated control, indicating an inability to prevent both *S. aureus* and *P. aeruginosa* biofilm growth in this mixed-species in vitro assay.

Table 1 presents the log CFU reduction values compared with untreated controls for the mixed-species biofilm prevention assay. Only four of the eight products were able to inhibit growth of *S. aureus* in the mixed-species biofilm prevention assay with CMC-1.2% Ag, Fish Skin, PU Foam-Ag Salt, and Poly-Sheet Metallic Ag unable to prevent the biofilm growth of *S. aureus* in a statistically significant manner compared with the control. Six of the eight products tested were able to inhibit biofilm growth of *P. aeruginosa* in a statistically significant manner compared with the control. Fish Skin, and PU Foam-Ag Salt were the only products unable to inhibit *P. aeruginosa* biofilm.

### 2.2. Efficacy of Wound Care Products in Preventing the Formation of New Biofilm in a CDFR Determined by CSLM Imaging

To enable for nondestructive, in situ microscopic evaluation of the biofilm matrix and embedded bacteria, the membrane/product biofilm containing pairs from the CDFR assays were evaluated via CSLM. The CSLM assay, which is a multi-step, and technically challenging assay involving staining, cryo-embedding, sectioning, and microscopy, required several experimental runs of CDFR/CSLM to obtain the appropriate images encompassing the cross-sectioned membrane–biofilm–product complex. The nature of some of the products tested also introduced additional difficulties to the CSLM process. For example, Collagenase was hard to slice through when sectioning, resulting in shattering of the gel in several repeated experiments. Once hydrated, CMC-Cellulose-1.7% Ag experienced cryo-embedding issues due to its thickness. Additionally, in some cases, the applied dressing products such as Poly-Sheet Metallic Ag tended to curl up and separate from the biofilm during sectioning.

**Figure 3:**
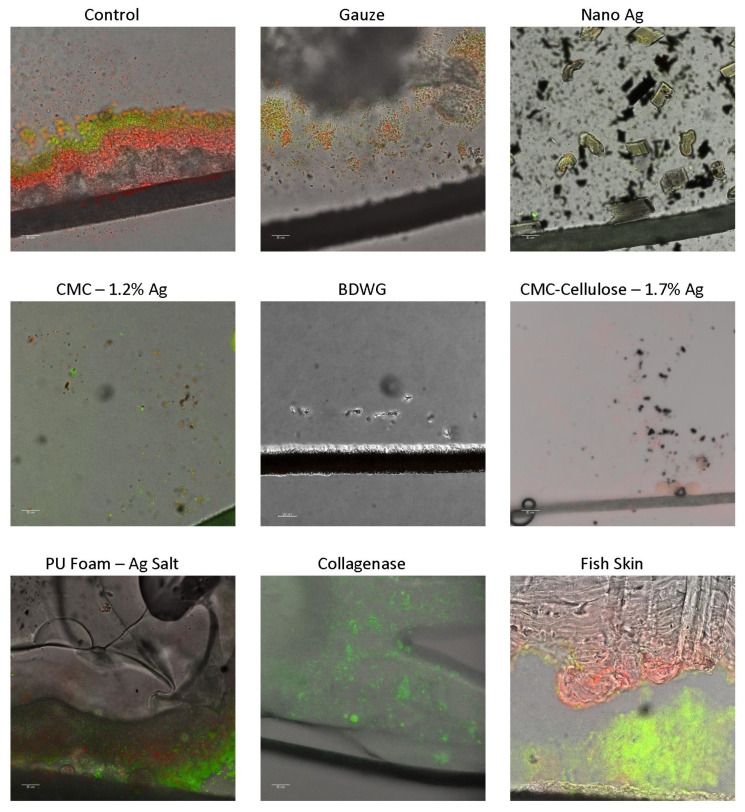
CSLM images of LIVE/DEAD™ stained cryosections from membrane/dressing pairs after the 24 h treatment period overlaid with transmitted light images. The black line near the bottom of the images is the biofilm support membrane.

Figure 3 shows CSLM imaging of cryosections of BacLight™ LIVE/DEAD™ stained membrane/dressing pairs overlaid with transmitted light images. The BacLight kit used to differentiate live and dead cells is composed of two nucleic acid stains: SYTO™9 and propidium iodide. SYTO 9 penetrates all bacterial membranes and stains the cells green, while propidium iodide only penetrates cells with damaged membranes, and the combination of the two stains produces red fluorescing cells.

All products were tested for biofilm prevention efficacy in the CDFR with the LIVE/DEAD CSLM assay. Although Poly-Sheet Metallic Ag was evaluated, it is not depicted due to poorly captured images associated with repeated cryo-embedding and sectioning issues. The LIVE/DEAD CSLM images determined that BDWG, Nano Ag and CMC-Cellulose-1.7% Ag dressings had superior biofilm growth prevention efficacy, as exhibited by the lack or substantially reduced detection of live green fluorescing bacteria within these biofilm cross-sectioned samples. Collagenase and CMC-1.2% Ag had reduced but detectable green fluorescing bacteria, while the untreated control, gauze, PU Foam-Ag Salt, and Fish Skin treated samples had significant detectable live bacteria in the biofilm. The LIVE/DEAD stained results generally correlated with the viable plate counts in the biofilm prevention CDFR assays.

**Figure 4:**
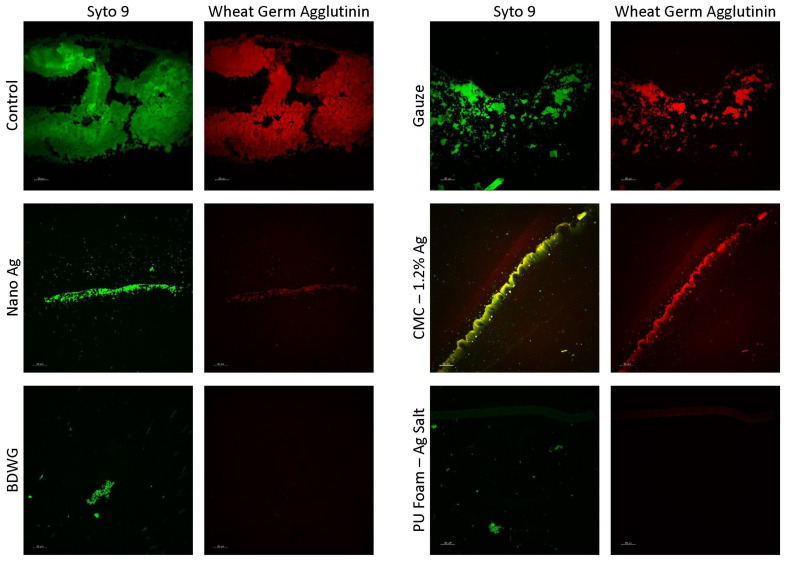
CSLM images of cryosections from membrane/dressing pairs after the 24 h treatment period stained with SYTO™ 9 and Texas Red^®^ conjugated Wheat Germ Agglutinin (WGA).

Figure 4 shows CSLM images of cryosections from membrane/dressing pairs after the 24 h treatment period, stained with SYTO™ 9 and Texas Red^®^ conjugated Wheat Germ Agglutinin (WGA). Rather than testing all products with SYTO 9/WGA, representative products with high, medium, and low or no staining in the BacLight LIVE/DEAD assay were evaluated in the SYTO 9/WGA assay. In this assay, SYTO 9 stains both Gram-positive and Gram-negative bacteria nucleic acid (green fluorescence), while WGA binds to poly-N-acetylglucosamine (PNAG) residues present in the typical EPS (red fluorescence). WGA staining in this assay was primarily bacterial cell-associated carbohydrate residues, which accounts for the correlation of SYTO 9 and WGA staining. All the tested treatments had less DNA and carbohydrate compared with the untreated and gauze controls, with BDWG and PU Foam-Ag Salt having the least detected fluorescence among the products tested. The SYTO 9/WGA staining correlated well with both the LIVE/DEAD staining and viable plate counts for majority of the products tested. The exception was the PU Foam-Ag Salt which appeared to show significantly reduced staining with SYTO 9/WGA but ample presence of live bacteria in the viable plate counts and LIVE/DEAD staining, indicating discrepancies between the assays. This discrepancy was attributed to technical staining difficulties with SYTO 9/WGA associated with loss of membrane/product during the CSLM process rather than an inhibitory mode of activity of PU Foam-Ag Salt in the CDFR SYTO 9/WGA assay.

### 2.3. Efficacy of Wound Care Products in Treating Established Mixed-Species DFR Biofilm by Viable Plate Count

**Figure 5:**
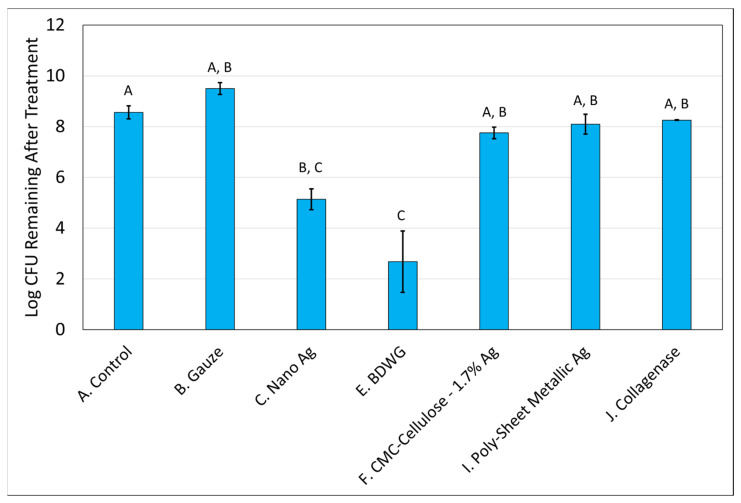
Log CFU /cm^2^ of *S. aureus* remaining in mixed-species mature biofilms after 24 h of treatment. If treatments have the same letter listed above the column, they are not statistically different at *p* = 0.05. The error bars depict the standard error of mean.

**Figure 6:**
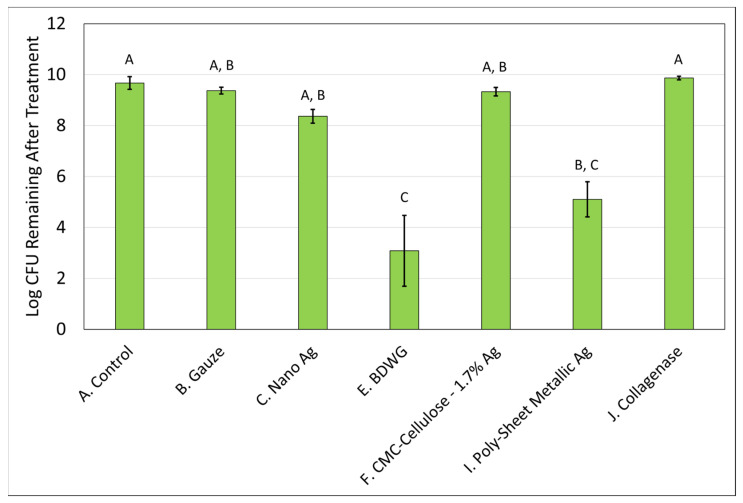
Log CFU/cm^2^ of *P. aeruginosa* remaining in mixed-species mature biofilms after 24 h of treatment. If treatments have the same letter listed above the column, they are not statistically different at *p* = 0.05. The error bars depict the standard error of mean.

Biofilm treatment/elimination was evaluated using the DFR model against mature mixed-species biofilms of *S. aureus* and *P. aeruginosa*. Only those products that demonstrated efficacy in the biofilm prevention CDFR assay against both bacteria were tested. The exception was Poly-Sheet Metallic Ag which only showed efficacy against *P. aeruginosa* in the biofilm prevention assay but was tested in the biofilm treatment DFR assays due to the difficulties encountered in evaluating the product in the biofilm prevention CDFR/CSLM/LIVE/DEAD assay. Mixed-species biofilms were grown on hydroxyapatite-coated slides for three days prior to treatment. The treatments were then applied for 24 h. Figure 5 and Figure 6 demonstrate the recovered log CFU/cm^2^ for *S. aureus* and *P. aeruginosa*, respectively from established biofilms either left untreated (control), treated with saline soaked gauze, or treated with wound care products for 24 hrs. Nano Ag and BDWG were efficacious at treating *S. aureus* entrenched mature biofilm in a statistically significant manner compared with the untreated control. CMC-Cellulose-1.7% Ag, Poly-Sheet Metallic Ag and Collagenase were ineffective at treating mature biofilm entrenched *S. aureus* in this in vitro mature biofilm treatment assay. BDWG and Poly-Sheet Metallic Ag were efficacious at treating *P. aeruginosa* entrenched mature biofilm in a statistically significant manner compared with the untreated control. Nano Ag, CMC-Cellulose-1.7% Ag, and Collagenase were ineffective at treating mature biofilm entrenched *P. aeruginosa* in this in vitro mature biofilm treatment assay.

Table 2 presents the log CFU reduction values compared with untreated controls for the mixed-species mature biofilm treatment assay. Only Nano Ag and BDWG were able to treat *S. aureus* in a mature biofilm in a statistically significant manner compared with the control, whereas BDWG and Poly-Sheet Metallic Ag were able to treat *P. aeruginosa* mature biofilm in a statistically significant manner compared with the control. As shown in Table 2, BDWG was the only product that exhibited greater than 2-log reductions for both microorganisms (5.88 logs for *S. aureus* and 6.58 logs for *P. aeruginosa*) and exhibited a statistically significant reduction of viable bacteria in a mature biofilm when compared with untreated control, indicating broad spectrum antimicrobial, and mature biofilm treatment efficacy.

## 3. Discussion

Infections caused by antibiotic-resistant bacteria were among the leading causes of death in 2019, with an estimated 4.95 million people dying from illnesses in which antimicrobial resistance (AMR) played a part, and an estimated 1.27 million of those deaths occurring as a direct result of AMR [44]. The presence of biofilm with its inherent AMR characteristics is medically recognized as a leading cause of chronicity of infections, with the Center for Disease Control and Prevention (CDC) estimating that biofilms are responsible for over 65% of all chronic bacterial infections, while the National Institutes of Health (NIH) estimating it at around 80% of microbial infections [7,8].

Bacteria can rapidly form a biofilm on wound surfaces and become tolerant to many traditional antimicrobial treatments through various mechanisms, presenting significant obstacles to clinical intervention of microbial colonization and infection [9,10,11,12,13,14,15,16,17,18]. The AMR develops over time, resulting in mature biofilms that are much more treatment resistant than newly formed biofilms [45,46,47,48]. Most in vitro studies evaluating the efficacy of wound treatment products against biofilms have used single-species biofilms. However, most chronic wound biofilms are polymicrobial, with *S. aureus* and *P. aeruginosa* often the most prevalent species [37,39,40].

This study was designed to evaluate both the anti-biofilm preventative and treatment capacity of commonly used wound care products against mixed-species (*S. aureus/P. aeruginosa*) biofilms in in vitro settings. Wound dressings impregnated with silver are widely used in wound management. To reflect this usage, five commercially available wound dressings with varied forms and concentrations of silver in varied dressing formats were evaluated. Additionally, a Collagenase enzymatic debridement product, an antimicrobial wound gel with EPS disruption capacity (BDWG) and a Fish Skin substitute lacking antimicrobial and anti-biofilm activity were also evaluated with the intent to explore the spectrum of antimicrobial and EPS disruption capacity in commercially available wound care products. Evaluation of the literature has determined that investigations of this combination of wound care products in both a biofilm prevention and mature biofilm treatment mixed-species biofilm CDFR and DFR model, respectively, have not yet been reported.

A mixed-species (*S. aureus* and *P. aeruginosa*) biofilm prevention assay was evaluated by applying the wound care products to the test surface immediately after mixed-species bacterial inoculation but before the bacteria had time to develop biofilm characteristics. As expected, products lacking antimicrobial agents (Gauze, Fish Skin) behaved as “no-treatment” controls. For *S. aureus* biofilm inhibition, the silver containing products Nano Ag and CMC-Cellulose-1.7% Ag and the non-silver-based products Collagenase and BDWG had the most effective results. The silver-based products tested were generally more successful at *P. aeruginosa* inhibition compared with *S. aureus* inhibition in the mixed-species biofilm. Silver-based products are known to vary in their effectiveness against bacteria depending on the form, concentration, and release of silver from the dressing, with reports of silver tending to be more effective against Gram negative bacteria compared with Gram positive bacteria [49,50,51,52,53,54,55]. Evaluation of biofilm inhibition efficacy of the tested products using LIVE/DEAD and SYTO 9/WGA staining generally indicated correlations between viable plate counts and CSLM assays. Biofilm prevention capabilities of silver-containing products in this study were also reported in single species biofilm prevention models [56]. Silver-containing dressings absorb wound exudates and associated microorganisms into the dressings to kill the absorbed microorganisms and/or release active silver ions from the dressing into the wound bed. The silver ions either in the dressing or the wound bed bind to proteins and nucleic acids and impede the metabolic and replicative capability of the bacteria [57]. The Collagenase product cleaves denatured collagen at seven specific sites along the denatured collagen strand. It is feasible that in an immature and thin biofilm, the Collagenase is able to access and cleave bacterial collagen structures in the EPS to delay but not completely inhibit biofilm maturation. BDWG is an antimicrobial wound gel composed of a “pH buffer system and benzalkonium chloride surfactant, which destabilizes the biofilm matrix through the chelation of calcium and removes proteins from bacterial membranes causing cell lysis” [58]

Compared with biofilm prevention, treatment of mature biofilms presents a much more significant challenge [45,46,47,48]. The DFR method used in this study is an American Society for Testing and Materials (ASTM)-approved method shown to produce biofilms with antibiotic tolerant characteristics similar to a porcine skin explant wound model, a mouse surgical excision wound model, and human clinical data [25,35,47,59]. Primarily, treatments that were efficacious against both bacteria in the mixed-species biofilm prevention assay were subsequently tested in the mixed-species mature biofilm treatment assay. Fewer products were efficacious at treatment of an established biofilm. Despite its theoretical capacity to impact EPS by degrading denatured collagen at several sites, Collagenase appeared unable to treat/disrupt the mixed-species biofilm in this in vitro setting. Nano Ag exhibited significant log reductions of *S. aureus,* which was consistent with prior publications [52,53], but was not efficacious against established *P. aeruginosa*, while Poly-Sheet Metallic Ag with its much lower concentration of silver (0.16 mg/in^2^ of total silver [60]), was only efficacious against *P. aeruginosa* in the biofilm treatment assays. BDWG designed to mechanistically disrupt EPS as well as lyse a broad spectrum of bacteria was the most effective wound care product tested in this study, with statistically significant mature biofilm treatment capability against both bacterial species.

The main goal of this work was to gather in vitro data on the efficacy of various wound care products (commercially available at the time the study was run) against mixed species of bacteria embedded in newly developing and mature biofilms. Using established in vitro biofilm assays, differentiation in the performance of wound care products with just antimicrobial technology (silver-based products) versus antimicrobial with EPS-dissolving technology (BDWG) was clearly evident. Although these in vitro assay results may not necessarily translate to in vivo efficacy, the data may be considered as a foundation to evaluating these products in other in vitro biofilm assays, ex vivo assays, biofilm-based animal models and human clinical trial settings.

The primary limitation of this study, as in most in vitro studies, is that no host components were included in the test systems. Continued and in-depth investigations are crucial to determining biofilm prevention and biofilm treatment capabilities of wound care products in use. Understanding which products specifically impact the protective EPS biofilm matrix could enable informed decisions on treatment of biofilm in wounds and potentially alleviate the significant morbidity and mortality associated with biofilm-related complications.

## 4. Materials and Methods

To aid identification, the study assigned an alpha character for each control and product tested (A through J).

Controls and Test Products:

A. Control: Mixed species inoculate left untreated.
B. Gauze: Saline saturated gauze was used to simulate sham treatment.
C. Nano Ag: An antimicrobial barrier dressing with a nanocrystalline coating of silver that rapidly kills a broad spectrum of bacteria in as little as 30 min. It consists of three layers: an absorbent inner core sandwiched between outer layers of silver coated, low adherent polyethylene net. Nanocrystalline Silver protects the wound site from bacterial colonization while the inner core helps maintain a moist wound environment.
D. CMC-1.2% Ag: The product is a silver-impregnated, antimicrobial, absorbent, sterile, non-woven hydroentangled dressing comprised of Hydrofiber (sodium carboxymethylcellulose). The dressing contains 1.2% *w*/*w* ionic silver. The silver in the dressing kills a broad spectrum of wound bacteria.
E. BDWG: The product is an antimicrobial wound gel made from citric acid (3.41%) sodium citrate (3.57%), benzalkonium chloride (0.13%), polyethylene glycol, and water buffered to a pH of 4 at an osmolarity of 2330 mOsm/L. The gel provides wound management by maintaining a moist wound environment conducive to wound healing. While in place, the gel can chelate metal ions from EPS causing its disruption and remove proteins from bacterial cell membranes causing their lysis.
F. CMC-Cellulose-1.7% Ag: The product is a non-woven dressing made of sodium carboxymethylcellulose (CMC), cellulose fibers, and silver oxysalts (0.2 mg Ag/cm^2^) (1.7 wt./wt.). The dressing reportedly kills at least 99.999% of a broad spectrum of bacteria, kills bacteria within a biofilm, and prevents biofilm reformation.
G. Fish Skin: The product is composed of intact fish skin and FDA coded as a skin substitute. The intact decellularized fish skin is used for the management of chronic wounds such as diabetic wounds, pressure ulcers, vascular ulcers, and draining wounds. The fish skin sheets contain fat, protein, elastin, glycans, and other natural skin elements.
H. PU Foam-Ag Salt: The product is an absorbent dressing made from Polyurethane foam. The outer surface of the foam is bonded to a vapor-permeable PU membrane and contains a silver salt that disperses into the wound fluid and is designed for the management of low to moderately exuding wounds. It may be used on infected wounds. The product has fast (from 30 min, in vitro), sustained (up to 7 days, in vitro), and broad range antimicrobial action (in vitro).
I. Poly-Sheet Metallic Ag: The product is a thin-film polymeric sheet composed of polyelectrolyte and polyvinyl alcohol containing ionic and metallic silver. The nanofilm matrix contains a low level of ionic and metallic silver (<25 µg/sq cm) to prevent microbial contamination and colonization of the matrix.
J. Collagenase: The product is an enzymatic debridement agent composed of an exogenous bacterial collagenase derived from fermentation by *Clostridium histolyticum*, with a pH of 6–8. The mechanism of action involves impacting necrotic tissue by cleaving at multiple sites of denatured collagen molecules and effectively removing barriers to healing, enabling wound progression by creating polypeptide bioactive byproducts.

Bacterial Strains and Media:

Test bacteria included two clinical isolates: methicillin-resistant *Staphylococcus aureus* (MRSA, MBL Strain 10943) and *Pseudomonas aeruginosa* (MBL Strain 215), which were initially obtained from the Southwest Regional Wound Care Center in Lubbock, TX. These strains have been used in several wound and biofilm-related studies, are maintained as frozen stock cultures at −80 °C and are available from the Center for Biofilm Engineering (CBE).

Inocula were grown overnight from frozen stock cultures in 10%-strength brain-heart infusion broth (10%-BHI, Difco™ 237200, Becton Dickenson, Sparks, MD, USA).

Prevention of Biofilm Formation using CDFR model:

Prevention of biofilm formation was evaluated using the CDFR model, which was developed to mimic the wound environment and evaluate wound dressings [36]. In this model, biofilms are grown on microporous membranes with a continuous supply of nutrients from beneath. Briefly, 2.5 cm diameter absorbent pads (AP1002500, Millipore Sigma, Burlington, MA, USA) were attached to the centers of glass microscope slides (48300-047, VWR International) and placed in a Drip Flow Biofilm Reactor^®^ (DFR 100-6, Biosurface Technologies Corp., Bozeman, MT, USA), which was then sterilized by autoclave. UV-sterilized, 1.3 cm diameter, 0.2 µm pore-size, polycarbonate membranes (GE Water & Process Technologies, Trevose, PA) were then placed on the absorbent pads. The *P. aeruginosa* culture was diluted 1:10 with phosphate-buffered saline (PBS) and mixed 1:1 with the MRSA culture. Ten µL of the mixture was applied to the centers of the membranes, and after 15 min of drying, treatments were applied to the top of the membrane. For gels, 0.5 mL was applied evenly over the membrane using a syringe. For dressings, a 2.5 cm × 2.5 cm^2^ was placed over the membrane. The flow of growth medium (10%-BHI) was then supplied at a rate of 5 mL/hr. per channel and the CDFR was incubated at 33 °C for 24 h. After incubation, each membrane/biofilm sample was placed in 10 mL of double-strength Dey-Engley neutralization broth (2X-DE, Becton, Dickinson, and Company, Sparks, MD) in a 50 mL conical tube (Falcon, Corning, NY) to neutralize the treatments. The tubes were then vortexed at high speed for 30 s, sonicated at 60 kHz in an ultrasonic cleaner (Model CSU3HE, Tuttnauer, Hauppauge, NY), and then vortexed again for 30 s to produce a biofilm suspension. At least three independent experiments were run for each treatment.

Plate Count Methodology:

After biofilm growth in the CDFR or DFR assay, samples were then recovered and neutralized with Dey-Engley neutralization broth (as described above in the Prevention of Biofilm Formation methods) to stop any remaining activity. The biofilm suspensions were serially diluted 10-fold using sterile PBS, and the dilutions were plated for selective counts on Pseudomonas Isolation Agar and Staphylococcus Medium 110 (Becton, Dickinson, and Company, Sparks, MD, USA) using spread-plate and drop-plate methods. After 24–48 h of incubation at 37 °C, the plates were counted, and the log colony-forming units per membrane (Log CFU/membrane) were calculated.

Confocal Scanning Laser Microscopy (CSLM):

For CSLM, the product/membrane pairs were removed from the reactor, and either stained with the BacLight™ LIVE/DEAD™ Bacterial Viability Kit (Life Technologies, Carlsbad, CA) using 0.5 µL/ mL of the SYTO™ 9 component and 1.5 µL/ mL of the propidium iodide component (final concentrations 1.67 and 30.0 µM, respectively) or left unstained. The LIVE/DEAD™ staining was performed in the dark for 10 min at room temperature. The samples were then cryo-embedded in Optimum Cutting Temperature compound (OCT, Tissue-Tek^®^, Fisher Healthcare, USA) and stored at −80 °C. Thin sections (10 µm) were cut at −20 °C using a Leica CM 1850 Cryostat (Leica, Wetzlar, Germany) and placed on glass microscope slides. Thin sections from the unstained samples were stained with a solution containing 1.67 µM SYTO™ 9 and 125 µg/ mL of Texas Red^®^ conjugate of wheat germ agglutinin (WGA, Life Technologies, Carlsbad, CA). Imaging was performed with a Leica SP5 confocal scanning laser microscope using a 63× water-immersion objective. For LIVE/DEAD™-stained samples, an excitation wavelength of 488 nm, and emission wave lengths of 500–550 nm and 600–650 nm were used. For the SYTO™ 9/WGA-stained samples, excitation wavelengths of 488 nm and 561 nm and emission wavelengths of 500–550 nm and 600–650 nm were used. Transmitted light images were also collected. The images were processed using Imaris^®^ Oxford Instruments, Abingdon UK) and Metamorph^®^ (Molecular Devices, San Jose, CA).

Treatment of Mature Biofilm using DFR model:

Treatments that were effective in preventing biofilm formation were further evaluated for efficacy against mature *S. aureus* and *P. aeruginosa* mixed-species biofilms established using the Drip Flow Biofilm Reactor^®^ (DFR 100-6, Biosurface Technologies Corp., Bozeman, MT, USA) equipped with hydroxyapatite-coated glass slides. The DFRs were placed in a horizontal position and inoculated with 1.0 mL of the mixed-species inoculum, as described above for the CDFR experiments. The DFRs were then inclined to a 10° angle, and the flow of growth medium (10%-BHI) was supplied at a rate of 10 mL/hr. per channel for three days at 33 °C. For treatment, the flow was halted, the DFRs were placed in a horizontal position, and the gels or products were applied. For the gels, 3.0 mL was applied evenly over the slide using a syringe. Dressings were cut precisely to match the glass slide and placed over biofilm. The DFRs were then incubated for 24 h without flow. The slides were then removed from the DFRs, scraped, rinsed with 10 mL 2X-DE into 50 mL conical vials, and placed in the vial. The vial was then vortexed–sonicated–vortexed, and plate counts were performed, as described in the plate count methodology. At least three independent experiments were run for each treatment.

Statistical Design

Log reduction (LR) was calculated relative to an untreated control membrane in each experiment. At least three independent experiments were conducted for each treatment in the prevention of biofilm formation assay using the CDFR model and in the treatment of mature biofilm assay using the DFR model. LR mean and standard deviations were calculated from individual experimental values. All individual LR values for each group were compared by ANOVA and Tukey’s post hoc test, and results were deemed significant at a *p*-values of <0.05 (Minitab 21.1.0).

## 5. Conclusions

In this in vitro study, several commercially available wound care products with varying mechanisms of action were tested for mixed-species biofilm prevention and treatment efficacy. The silver containing wound care products Nano Ag and CMC-Cellulose 1.7% Ag, the Collagenase product and the antimicrobial gel-BDWG exhibited statistically significant efficacy in preventing new biofilm formation by both *S. aureus* and *P. aeruginosa*. However, the antimicrobial gel BDWG, was the only product that demonstrated statistically significant efficacy in treating mature biofilms of both bacterial species when compared with untreated controls.

## Figures and Tables

**Table 1:** Compiled Log CFU reduction of *S. aureus* and *P aeruginosa* species in comparison with untreated controls in a mixed-species CDFR biofilm prevention assay.

		B. Gauze	C. Nano Ag	D. CMC-1.2% Ag	E. BDWG	F. CMC-Cellulose-1.7% Ag	G. Fish Skin	H. PU Foam-Ag Salt	I. Poly-Sheet Metallic Ag	J. Collagenase
Prevention of Mixed Species New Biofilm Formation	*S. aureus*	−0.96	6.94 (Y)	2.29	7.04 (Y)	6.72 (Y)	−0.69	−0.75	0.47	3.47 (Y)
	*P. aeruginosa*	−0.48	8.13 (Y)	4.23 (Y)	7.75 (Y)	9.13 (Y)	−0.61	0.38	4.03 (Y)	4.89 (Y)

All individual Log Reduction (LR) values for each group were compared by ANOVA and Tukey’s post hoc test, and results were deemed significant at a *p*-values of <0.05 (Minitab 21.1.0). Log CFU reduction values followed by (Y) indicates a statistically significant reduction when compared with untreated controls by *t*-test (*p* < 0.05).

**Table 2:** Log CFU reduction of *S. aureus* and *P aeruginosa* species in comparison with untreated controls in a mixed-species DFR mature biofilm treatment assay.

		B. Gauze	C. Nano Ag	D. CMC-1.2% Ag	E. BDWG	F. CMC-Cellulose-1.7% Ag	G. Fish Skin	H. PU Foam-Ag Salt	I. Poly-Sheet Metallic Ag	J. Collagenase
Treatment of Mature Mixed-Species Biofilm	*S. aureus*	−0.94	3.42 (Y)	NT	5.88 (Y)	0.81	NT	NT	0.46	0.3
	*P. aeruginosa*	0.3	1.3	NT	6.58 (Y)	0.34	NT	NT	4.57 (Y)	−0.2

All individual LR values for each group were compared by ANOVA and Tukey’s post hoc test and results were deemed significant at a *p*-value of <0.05 (Minitab 21.1.0). Log CFU reduction values followed by alpha character (Y) indicates statistically significant reduction when compared with untreated controls by *t*-test (*p* < 0.05). NT = Product not tested in the DFR mature biofilm treatment assay.

## Data Availability

The raw data presented in this study are available on request from the corresponding author. The data are not publicly available due to Next Science’s data sharing quality guidelines.
